# Spirituality and Religiosity in End-of-Life Decision-Making in Intensive Care Units: A Systematic Review of Preferences, Ethical Framing, and Meaning-Making

**DOI:** 10.7759/cureus.115135

**Published:** 2026-08-25

**Authors:** Dimitrios G Apostolakis, Nikiforos V Angelopoulos, Spiros Georgakis, Fotios Tatsis, Foteini Veroniki, Konstantinos Stamatis, Georgios Papathanakos, Mary Gouva, Vasilios Koulouras

**Affiliations:** 1 Faculty of Medicine, School of Health Sciences, University of Ioannina, Ioannina, GRC; 2 Psychiatry, University of Thessaly, Larisa, GRC; 3 Intensive Care Unit, University Hospital of Ioannina, Ioannina, GRC; 4 Scientific Laboratory of Psychology and Person-Centered Care, University of Ioannina, Ioannina, GRC

**Keywords:** end-of-life decision-making, ethical decision-making, intensive care units, life-sustaining treatment, palliative care, religion, spirituality, surrogate decision-making

## Abstract

End-of-life decision-making in intensive care units (ICUs) is a complex and ethically challenging process shaped not only by clinical factors but also by patients' values, cultural contexts, and spiritual or religious beliefs. Although spirituality and religiosity are increasingly recognized as influential in decisions regarding life-sustaining treatment, their role has not been comprehensively synthesized within ICU settings. This systematic review, conducted in accordance with Preferred Reporting Items for Systematic Reviews and Meta-Analyses (PRISMA) guidelines, examined the influence of spirituality and religiosity on end-of-life decision-making among adult ICU patients, surrogate decision-makers, and healthcare professionals. Electronic searches of PubMed/MEDLINE, Scopus, and Web of Science identified eligible quantitative, qualitative, and mixed-methods studies, and findings were synthesized narratively because of methodological heterogeneity. Eight studies met the inclusion criteria. Overall, higher levels of religiosity were consistently associated with a stronger preference for aggressive life-prolonging interventions and a lower likelihood of treatment limitation. Spiritual and religious beliefs also influenced clinicians' ethical perspectives, particularly regarding the distinction between withholding and withdrawing life-sustaining treatments, while playing a central role in surrogate decision-making by shaping interpretations of illness, hope, and acceptable care. At the same time, spirituality demonstrated a multidimensional role, facilitating acceptance of death, promoting support for palliative care in some contexts, and contributing to meaning-making during the end-of-life process. Cultural and contextual factors moderated these relationships, and the provision of spiritual care by healthcare professionals was associated with less aggressive end-of-life care. These findings indicate that spirituality and religiosity exert a substantial yet context-dependent influence on end-of-life decision-making in ICUs. Integrating spiritual assessment and culturally sensitive spiritual care into routine clinical practice may enhance shared decision-making and improve alignment between treatment decisions and patients' values, preferences, and goals of care.

## Introduction and background

End-of-life decision-making in intensive care unit (ICU) settings represents one of the most complex and ethically challenging aspects of modern healthcare. Decisions regarding the initiation, continuation, withholding, or withdrawal of life-sustaining treatments often occur under conditions of clinical uncertainty, emotional distress, and time pressure, involving not only patients but also family members and healthcare professionals [1,2]. These decisions are influenced by a wide range of factors, including medical prognosis, patient preferences, cultural norms, and ethical frameworks.

Among these factors, spirituality and religiosity have emerged as important yet insufficiently understood determinants of end-of-life decision-making. Although often used interchangeably, spirituality and religiosity represent distinct but overlapping constructs. Spirituality is generally defined as a broader concept encompassing individuals’ search for meaning, purpose, and connection, which may or may not be linked to formal religious beliefs, whereas religiosity typically refers to structured beliefs, practices, and affiliations associated with organized religion [3,4]. Both constructs can shape how individuals interpret illness, suffering, and death, and may therefore influence preferences and decisions at the end of life.

Previous research has suggested that spirituality and religiosity may affect end-of-life care in multiple ways. Higher levels of religiosity have been associated with a greater likelihood of pursuing life-prolonging treatments and lower utilization of palliative care services [5,6]. These patterns have often been attributed to beliefs in divine intervention, hope for recovery, or moral concerns regarding the limitation of treatment. At the same time, spirituality may provide a framework for acceptance of death, facilitate coping, and support decisions aligned with comfort-focused care [7,8]. These seemingly contrasting effects highlight the complexity of the relationship between spirituality, religiosity, and end-of-life decision-making.

In ICU settings, this complexity is further amplified by the involvement of multiple stakeholders, including critically ill patients, surrogate decision-makers, physicians, and nurses, each bringing their own beliefs, values, and experiences into the decision-making process [9]. Moreover, cross-cultural differences in religious traditions, societal values, and healthcare systems contribute to substantial variability in end-of-life practices across regions [10,11]. As a result, the influence of spirituality and religiosity cannot be understood in isolation but must be considered within broader cultural and clinical contexts.

Despite growing interest in this topic, the existing literature remains fragmented, with studies differing in populations, methodologies, and definitions of spirituality and religiosity. To date, there is a lack of comprehensive synthesis focusing specifically on ICU settings and examining how spirituality and religiosity influence attitudes, preferences, and clinical decisions related to end-of-life care.

Therefore, the aim of this systematic review is to examine the role of spirituality and religiosity in shaping end-of-life decision-making in ICU settings, including their influence on attitudes, treatment preferences, and actual clinical practices.

## Review

Methods

This systematic review was conducted to examine the influence of spirituality and religiosity on end-of-life decision-making in ICU and critical care contexts.

The eligibility criteria were defined based on the Population-Exposure-Outcome (PEO) framework. Studies were considered eligible if they involved adult populations (≥18 years), including critically ill patients, family members or surrogate decision-makers, and healthcare professionals, and addressed end-of-life decision-making within ICU or critical care settings, or examined decisions directly applicable to end-of-life care in critical care practice. Eligible studies examined spirituality and/or religiosity as a primary or secondary variable and reported outcomes related to end-of-life decision-making, including attitudes, preferences, or actual clinical decisions, such as do-not-resuscitate (DNR) orders, withholding or withdrawing life-sustaining treatment, and palliative care decisions. Quantitative, qualitative, and mixed-methods studies were eligible provided that they were published in peer-reviewed journals and written in English. Studies were excluded if they focused on pediatric populations, did not assess spirituality or religiosity in relation to end-of-life decision-making, or were editorials, commentaries, letters, case reports, or purely theoretical papers without empirical data. Studies not available in full text were also excluded.

A comprehensive literature search was conducted in PubMed/MEDLINE, Scopus, and Web of Science, with the final searches completed on January 15, 2026. Database-specific search strategies combined terms related to spirituality and religiosity, end-of-life decision-making, intensive and critical care, and adult populations, while excluding pediatric populations. No additional database filters or limits were applied. Manual screening of the reference lists of included studies did not identify any additional eligible studies. The complete database-specific search strategies, including the final search date and the number of records retrieved from each database, are provided in the Appendix.

All identified records were imported into reference management software (Zotero), and duplicate records were removed. The study selection process was conducted in two stages. Initially, titles and abstracts were screened for relevance. Subsequently, full texts of potentially eligible studies were assessed against the predefined inclusion and exclusion criteria. Screening was performed independently by two reviewers, and disagreements were resolved through discussion or consultation with a third reviewer.

Data extraction was carried out using a standardized data extraction form developed specifically for this review. Extracted information included study characteristics (author, year of publication, country), study design, population characteristics, measures of spirituality and/or religiosity, end-of-life decision-making outcomes, and key findings.

Methodological quality and risk of bias were assessed using the Joanna Briggs Institute (JBI) critical appraisal tools appropriate to each study design. Cohort studies were evaluated using the JBI Checklist for Cohort Studies, analytical cross-sectional studies using the JBI Checklist for Analytical Cross-Sectional Studies, and the qualitative study using the JBI Checklist for Qualitative Research. Appraisal judgments were used to identify study-specific methodological strengths and limitations and to inform the interpretation of the narrative synthesis; no numerical quality score or arbitrary cut-off was applied. Critical appraisal was performed independently by two reviewers, with disagreements resolved through discussion and consensus.

Because of substantial heterogeneity in study populations, designs, measures of spirituality and religiosity, and end-of-life outcomes, quantitative pooling was considered inappropriate and no meta-analysis was performed. Accordingly, no pooled effect estimates, P-values, or 95% confidence intervals were calculated at the review level. Statistical estimates from individual studies were interpreted as reported in the original publications. A narrative synthesis was undertaken, with findings synthesized thematically according to associations between spirituality and religiosity and attitudes toward end-of-life care, preferences regarding life-sustaining treatment, and reported or observed clinical decision-making. Differences and similarities across patients, family members or surrogate decision-makers, and healthcare professionals were also examined.

This systematic review was not prospectively registered in PROSPERO, or another systematic review registry, and no formal review protocol was publicly deposited.

Results

Study Selection

**Figure 1:**
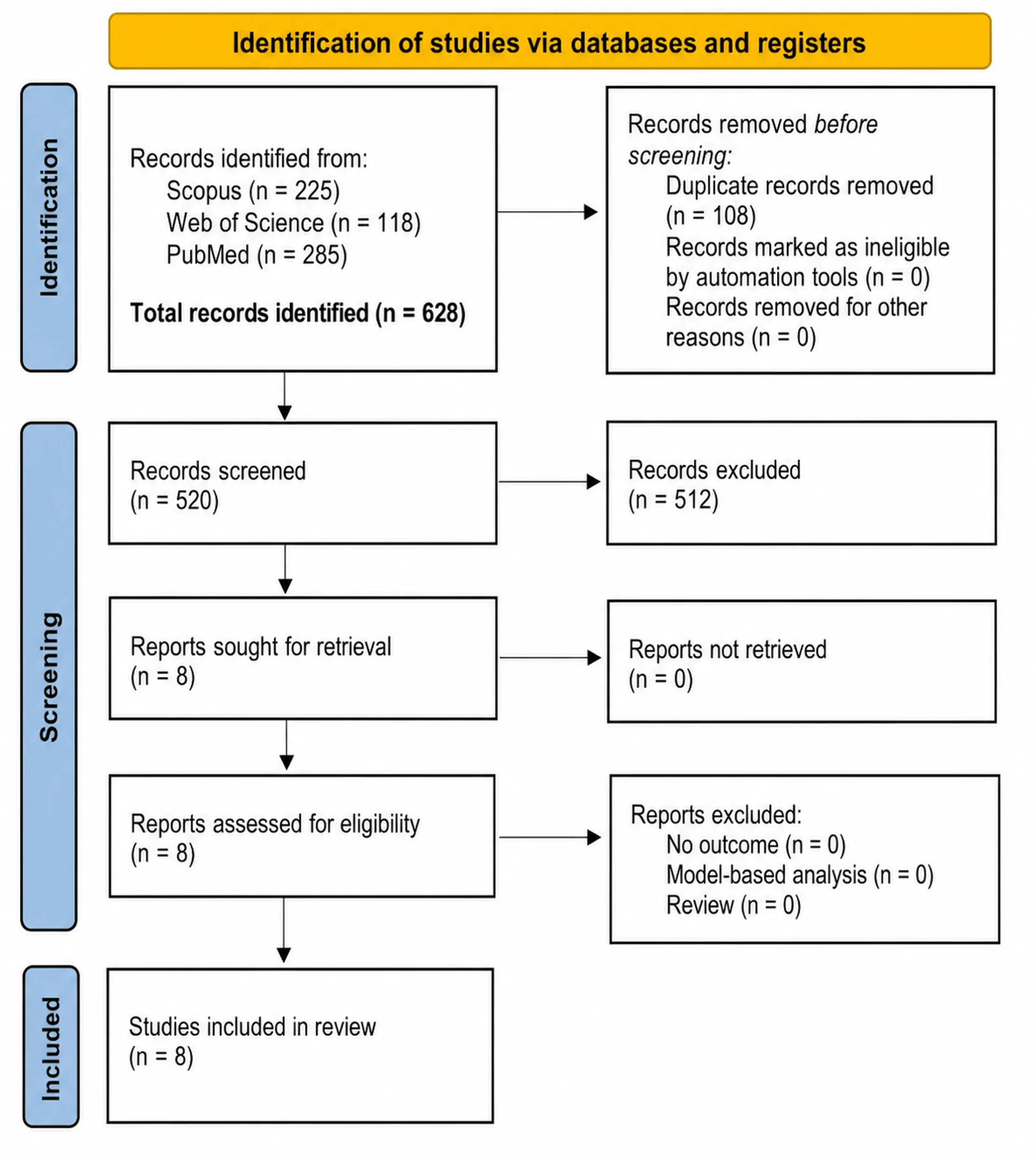
Preferred Reporting Items for Systematic Reviews and Meta-Analyses (PRISMA) flow diagram of the study selection process

The study selection process is presented in Figure 1. The database searches identified 628 records: 285 from PubMed/MEDLINE, 225 from Scopus, and 118 from Web of Science. After the removal of 108 duplicate records, 520 records were screened based on their titles and abstracts, of which 512 were excluded. Eight reports were sought for retrieval, and all were successfully retrieved and assessed for eligibility. No reports were excluded following full-text assessment, and eight studies were included in the final narrative synthesis.

Characteristics of the Included Studies

The characteristics of the included studies are summarized in Table 1. Overall, the eight studies comprised quantitative and qualitative designs conducted across diverse geographical settings, including the United States, Europe, Asia, and Greece.

**Table 1:** Characteristics of the included studies Abbreviations: RCOPE, Religious Coping Scale; ICU, intensive care unit; DNR, do-not-resuscitate

Study	Country	Population	Study design	Spirituality/religiosity measure	End-of-life outcomes	Key findings
Balboni et al., 2013 [12]	USA	343 patients with advanced cancer	Prospective cohort	Brief RCOPE, importance of religion, spiritual support (community and medical team)	Hospice use, ICU admission, aggressive interventions	High religious community support → less hospice, more aggressive care and ICU deaths; spiritual care by clinicians → less aggressive care
Cardoso et al., 2003 [13]	Portugal	175 ICU physicians	Cross-sectional survey	Religious affiliation (Catholic vs. atheist/agnostic)	DNR decisions, withholding/withdrawing treatment, family involvement	Religion influenced decision process: non-religious physicians involved families more; criteria for decisions remained similar
Chung et al., 2016 [14]	USA	1,156 physicians	Cross-sectional survey	Importance of religion, attendance, religious affiliation	Attitudes toward withdrawing vs withholding treatment	Higher religiosity → greater perception that withdrawal is ethically problematic; no effect on compliance with patient wishes
Geros-Willfond et al., 2016 [15]	USA	46 surrogate decision-makers	Qualitative study	Thematic analysis of religious beliefs	Decision-making role, DNR, withdrawal decisions	Religion guided decisions; belief in divine control sometimes delayed withdrawal, but also facilitated acceptance of death in some cases
Guidet et al., 2018 [16]	Europe (21 countries)	5,021 ICU patients ≥80 yrs	Prospective multicenter	Country-level religiosity	Withholding/withdrawing treatment	Higher religiosity associated with lower treatment limitation rates (more aggressive care)
Ntantana et al., 2017 [17]	Greece	149 physicians and 320 nurses	Cross-sectional	SpREUK scale (intrinsic religiosity)	Attitudes toward withdrawing treatment	Higher religiosity → lower willingness to withdraw life support (especially ventilation); differences between nurses and physicians
Phua et al., 2015 [11]	Asia (16 countries)	1,465 physicians	Multicenter survey	Religious affiliation	DNR decisions, withholding/withdrawing treatment	Religion influenced DNR decisions; non-religious physicians less likely to implement DNR; strong cultural variability
Schefold et al., 2023 [10]	Europe	4,592 ICU patients	Comparative cohort (Ethicus I and II)	Religious affiliation (patients and physicians)	Withholding vs. withdrawing treatment	Temporal changes in end-of-life practice were not fully explained by patient or physician religious affiliation.

The study populations encompassed critically ill patients, surrogate decision-makers, physicians, and nurses, reflecting the multidisciplinary and multi-stakeholder nature of end-of-life decision-making in ICU settings. Sample sizes ranged from small qualitative cohorts of surrogate decision-makers (n = 46) to large multicenter investigations involving several thousand participants. Spirituality and religiosity were assessed using diverse approaches, including validated instruments such as the Brief RCOPE (Religious Coping Scale) and SpREUK scales, self-reported measures of religious involvement (e.g., importance of religion and religious service attendance), and religious affiliation.

This methodological diversity provided a broad perspective on the ways spirituality and religiosity have been conceptualized and examined in relation to end-of-life decision-making across different clinical and cultural contexts.

Methodological Quality and Risk of Bias

The JBI critical appraisal identified methodological strengths across the included studies, alongside design-specific limitations that were considered when interpreting the evidence. The prospective cohort studies were strengthened by prospective data collection, large multicentre samples, clinically defined end-of-life outcomes, and adjustment for relevant covariates. Nevertheless, important limitations included residual confounding and indirect or incomplete assessment of religiosity, including the use of country-level measures or religious affiliation rather than individual multidimensional assessments of religiosity or spirituality.

The cross-sectional studies contributed evidence from national and multinational samples but were inherently limited in their ability to support causal inference. Recurring methodological concerns included reliance on self-reported attitudes or hypothetical decision-making scenarios, potential non-response or selection bias, heterogeneity in the operationalization of religiosity, and variation in the extent to which confounding factors were addressed. The qualitative study was strengthened by semi-structured interviews, iterative thematic analysis, multiple coders, and direct representation of participants’ perspectives, although researcher reflexivity and philosophical positioning were not fully reported.

No study was excluded on methodological grounds. The identified limitations were considered throughout the narrative synthesis, particularly when interpreting associations between spirituality or religiosity and end-of-life decision-making. Study-specific methodological strengths and limitations are summarized in Table 2.

**Table 2:** Critical appraisal of the included studies using JBI tools Appraisal approach: Critical appraisal was conducted using the Joanna Briggs Institute (JBI) checklist appropriate to each study design (cohort, analytical cross-sectional, or qualitative research). Appraisal findings were used to identify design-specific methodological strengths and limitations and were not converted into a numerical quality score. Abbreviations: ICU, intensive care unit; JBI, Joanna Briggs Institute Note: All eight studies were retained for the narrative synthesis.

Study	Study design	Methodological strengths	Main methodological limitations
Balboni et al., 2013 [12]	Prospective cohort	Prospective design; clinically documented end-of-life outcomes; multivariable adjustment for relevant covariates.	Spiritual support primarily assessed by self-report; incomplete data for a small proportion of participants; residual confounding cannot be excluded.
Cardoso et al., 2003 [13]	Cross-sectional national survey	National ICU physician sample; clearly defined study population; appropriate statistical comparisons.	Religiosity operationalized mainly as religious affiliation; self-reported attitudes and practices; limited multivariable control for confounding; potential non-response bias.
Chung et al., 2016 [14]	Cross-sectional national physician survey	Large national physician sample; assessment of several indicators of religious involvement; consideration of relevant professional and clinical characteristics.	Self-reported attitudinal outcomes; limited psychometric information for outcome items; potential non-response bias; cross-sectional design.
Geros-Willfond et al., 2016 [15]	Qualitative study	Semi-structured interviews; verbatim transcription; iterative thematic analysis; multiple coders; strong representation of participants’ voices.	Limited reporting of philosophical positioning and researcher reflexivity; potential participation bias; restricted geographic and religious diversity.
Guidet et al., 2018 [16]	Prospective multicentre cohort	Large multinational ICU cohort; high follow-up completeness; multilevel adjustment for patient- and country-level factors.	Religiosity assessed at country rather than individual level, creating a risk of ecological inference; participating ICUs may not fully represent national practice; patient and family preferences were not directly assessed.
Ntantana et al., 2017 [17]	Multicentre analytical cross-sectional study	ICU-specific multicentre sample; structured assessment of religiosity/spirituality; multivariable analysis.	Self-reported attitudes rather than observed clinical decisions; potential non-response bias; dichotomization of some continuous variables; cross-sectional design.
Phua et al., 2015 [11]	Multinational cross-sectional survey	Large multinational ICU sample; systematic questionnaire development; multilevel adjustment for physician-, ICU-, and country-level factors.	Religious affiliation used as a proxy for religiosity; self-reported or hypothetical outcomes; limited formal reliability testing; heterogeneous sampling methods and potential non-response bias.
Schefold et al., 2023 [10]	Comparative observational cohort	Large multicentre sample; consistent end-of-life definitions across study periods; statistical adjustment for clustering and relevant covariates.	Religious affiliation rather than strength of religiosity; substantial missing or unknown patient religious affiliation; incomplete participation of original ICU sites; observational design and selected analytic population.

Influence of Spirituality and Religiosity on End-of-Life Decision-Making

The thematic synthesis identified several recurring and interrelated patterns across the included studies, encompassing treatment preferences, ethical framing, surrogate decision-making, cultural and temporal influences, and the role of spiritual care. These themes are summarized in Table 3.

**Table 3:** Thematic synthesis of findings on spirituality/religiosity and end-of-life decision-making in ICU settings Abbreviations: DNR, do-not-resuscitate; ICU, intensive care unit Note: Associations summarized in this table should be interpreted in light of the observational and heterogeneous nature of the included evidence.

Theme	Description	Supporting studies	Key evidence
Preference for Life-Prolonging Care	Greater religiosity or religious/spiritual support was associated in some studies with greater use or preference for life-prolonging treatment and lower use of hospice or treatment limitation.	Balboni et al., 2013 [12]; Guidet et al., 2018 [16]	Religious-community spiritual support was associated with more intensive end-of-life care, while higher country-level religiosity was associated with lower rates of treatment limitation.
Reluctance to Withdraw Treatment	Religious engagement was associated with greater reluctance to withdraw life-sustaining treatment, particularly among healthcare professionals.	Chung et al., 2016 [14]; Ntantana et al., 2017 [17]	More religious clinicians were more likely to regard withdrawal as ethically problematic or to express lower willingness to withdraw particular forms of life support.
Ethical Framing of Withholding and Withdrawal	Religious characteristics were associated with how clinicians perceived the ethical distinction between withholding and withdrawing treatment.	Chung et al., 2016 [14]; Phua et al., 2015 [11]	Withdrawal was more often perceived as morally or psychologically distinct from withholding in some religious and cultural contexts.
Influence on the Decision-Making Process	Religion appeared to shape aspects of how decisions were made, including family involvement and DNR-related attitudes, without uniformly determining the underlying clinical criteria.	Cardoso et al., 2003 [13]; Phua et al., 2015 [11]	Religious affiliation was associated with selected features of decision-making and communication, with substantial variation across professional and cultural contexts.
Spirituality in Surrogate Decision-Making	Spiritual and religious beliefs provided frameworks through which surrogates interpreted illness, uncertainty, responsibility, and treatment limitation.	Geros-Willfond et al., 2016 [15]	Belief in divine control could contribute to reluctance to withdraw treatment, whereas other spiritual interpretations facilitated acceptance of death.
Dual Role of Spirituality	Spirituality could sustain hope and continuation of treatment but could also facilitate acceptance of dying and comfort-focused care.	Geros-Willfond et al., 2016 [15]; Balboni et al., 2013 [12]	The direction of the association depended on the meaning attributed to spiritual beliefs and on the relational and clinical context.
Cultural and Contextual Influences	Associations between religion and end-of-life decisions varied according to cultural, regional, and healthcare-system context.	Phua et al., 2015 [11]; Guidet et al., 2018 [16]; Schefold et al., 2023 [10]	Considerable variation was observed across countries and regions, indicating that religious variables cannot be interpreted independently of their sociocultural context.
Temporal Changes in End-of-Life Practice	Changes in European ICU end-of-life practices over time were not fully explained by patient or physician religious affiliation.	Schefold et al., 2023 [10]	Communication and family involvement changed substantially across study periods, while the contribution of religious affiliation remained complex and non-uniform.
Spiritual Care Provided by Clinicians	Clinician-provided spiritual care was associated with less aggressive end-of-life care in observational evidence.	Balboni et al., 2013 [12]	Spiritual care and end-of-life discussions were associated with greater hospice use and less intensive treatment, although causal inference is not possible.

Higher levels of religiosity and spiritual support were frequently associated with a greater preference for aggressive, life-prolonging care. In a large prospective cohort study, patients who reported strong spiritual support from religious communities were less likely to receive hospice care and more likely to undergo intensive interventions, including ICU admission and life-sustaining treatments [12]. Similarly, multicenter European data indicated that higher levels of societal religiosity were associated with lower rates of withholding or withdrawal of life-sustaining treatment [16].

At the clinician level, religiosity was found to influence both attitudes and clinical decision-making practices. Physicians with higher levels of religious engagement were more likely to perceive withdrawal of life-sustaining treatment as ethically problematic compared with withholding treatment, despite generally supporting patient autonomy [14]. Consistently, ICU professionals with higher intrinsic religiosity demonstrated a lower willingness to withdraw life-sustaining treatments, particularly mechanical ventilation [17].

Religiosity also appeared to shape the ethical framing of end-of-life decisions. In both Western and Asian contexts, clinicians frequently perceived withholding and withdrawing treatment as ethically distinct, with religious characteristics influencing these perceptions [11,14]. However, the effect of religiosity on actual clinical behavior was not uniform. In some cases, religious affiliation influenced the decision-making process-such as the degree of family involvement-rather than the fundamental criteria used to guide decisions [13].

Qualitative findings further highlighted the central role of spirituality in surrogate decision-making. Surrogates often relied on religious beliefs to interpret illness, guide decision-making, and cope with uncertainty. Belief in divine control over life and death was associated with reluctance to withdraw life-sustaining treatments in some cases, while in others, spirituality facilitated acceptance of death and support for palliative care decisions [15].

Importantly, the relationship between spirituality and end-of-life decision-making was not unidirectional. While religiosity was frequently associated with preferences for more aggressive care, it could also promote acceptance of end-of-life transitions, depending on individual beliefs and contextual factors [12,15]. Temporal changes in end-of-life practices were not fully explained by religious factors alone, indicating the relevance of broader cultural and healthcare-system characteristics [10].

Finally, one included study reported an association between clinician-provided spiritual care and end-of-life treatment patterns. Patients whose spiritual needs were addressed within clinical care were less likely to receive aggressive end-of-life interventions and more likely to receive palliative approaches [12].

Discussion

In ICU settings, end-of-life decisions are rarely made on purely clinical grounds. Rather, they emerge within broader frameworks of meaning through which patients, families, and clinicians interpret illness, suffering, and the prospect of death. The present systematic review demonstrates that spirituality and religiosity play a significant yet multifaceted role in these processes, influencing not only patients' and families' treatment preferences but also clinicians' ethical perceptions and clinical practices. This influence is neither uniform nor unidirectional; instead, it is shaped by the dynamic interaction of spiritual beliefs with cultural, relational, and contextual factors. Collectively, these findings suggest that spirituality and religiosity function less as static individual characteristics than as interpretive resources through which individuals negotiate uncertainty, assign meaning to critical illness, and approach end-of-life decisions.

One of the most consistent findings across the included studies is the association between higher levels of religiosity and a greater preference for aggressive, life-prolonging interventions. This is in line with previous research indicating that patients with stronger religious coping mechanisms are more likely to pursue intensive treatments near the end of life and less likely to utilize hospice or palliative care services [5,6]. While this association is often explained in terms of beliefs in divine intervention, hope for recovery, or the possibility of miracles, it may also be understood as reflecting a deeper psychological process. In conditions of existential threat, such as critical illness, the preservation of life may acquire a symbolic dimension [18], representing not only biological survival but also the maintenance of meaning, continuity, and relational bonds. In this sense, the pursuit of aggressive treatment may be interpreted not solely as a medical preference, but as an expression of an underlying difficulty in relinquishing the possibility of continuation.

These findings may also be interpreted in relation to psychological theories of mortality awareness and death acceptance. Terror Management Theory proposes that awareness of mortality can generate existential anxiety and increase reliance on cultural worldviews, self-esteem, and systems of meaning that provide a sense of continuity, value, and psychological security [19]. More recent applications of terror management theory to life-threatening illness and end-of-life care suggest that these processes may be particularly relevant when individuals are confronted not with abstract reminders of death but with the concrete possibility of dying [20,21]. From this perspective, religious and spiritual beliefs may function not simply as predictors of particular treatment preferences but as broader meaning systems through which mortality, uncertainty, and existential threat are interpreted and managed. Importantly, however, engagement with mortality does not necessarily result only in defensive responses. Experimental evidence indicates that heightened and deliberate mortality awareness may, under some conditions, reduce fear of dying and increase acceptance of dying, with meaning-related responses also varying according to religiosity [22]. These complementary perspectives may help explain why spirituality was associated in the reviewed literature both with continued pursuit of life-prolonging treatment and, in other contexts, with acceptance of dying and greater openness to comfort-focused care.

At the same time, the present review highlights that religiosity strongly shapes the ethical framing of end-of-life decisions, particularly the distinction between withholding and withdrawing treatment. This finding aligns with prior literature suggesting that clinicians frequently perceive withdrawal of life-sustaining treatment as more morally and psychologically burdensome than withholding it, despite their ethical equivalence in most bioethical frameworks [23]. Religious beliefs appear to intensify this distinction, potentially by reinforcing moral intuitions related to action versus omission, as well as concerns about responsibility for death. From a psychological perspective, this asymmetry may reflect a deeper discomfort with acts experienced as active participation in the ending of life, as opposed to those perceived as allowing death to occur. Such distinctions may also serve a defensive psychological function [24], helping clinicians and families manage feelings of guilt, responsibility, and moral conflict. Consequently, the ethical tension observed in these decisions may not be reducible to rational deliberation alone but may also be shaped by underlying emotional and symbolic processes.

Importantly, the findings also indicate that spirituality plays a central role in surrogate decision-making. Previous qualitative studies have similarly shown that family members often draw upon religious beliefs to interpret medical information, cope with uncertainty, and justify their decisions [25,26]. In some cases, belief in divine control over life and death may lead to resistance toward withdrawing life-sustaining treatment, while in others, spiritual frameworks may facilitate acceptance of death and support for palliative care. This dual role of spirituality underscores its complexity and suggests that its impact depends not only on the content of beliefs but also on how these beliefs are internally experienced and mobilized in moments of crisis. Spirituality may thus function both as a means of sustaining hope and as a pathway toward acceptance, allowing individuals to tolerate the limits of medical intervention and the inevitability of loss. This perspective is consistent with existential approaches emphasizing that the search for meaning may remain possible even in the presence of profound suffering and approaching death [18].

An important limitation of the existing evidence is that end-of-life decision-making is often represented through attitudes, treatment preferences, and clinical outcomes, whereas the lived experience of dying remains comparatively less visible. The qualitative evidence included in this review partially addresses this gap by illustrating how surrogate decision-makers use religious and spiritual beliefs to make sense of uncertainty, responsibility, impending loss, and the possibility of death. Nevertheless, first-person accounts from critically ill patients and frontline healthcare professionals remain underrepresented in the available literature. Greater attention to such narratives may deepen understanding not only of which end-of-life decisions are made, but also of how death is experienced, spoken about, feared, accepted, and given meaning within intensive care.

Another important finding is that associations involving religiosity varied across cultural and systemic contexts. Cross-national studies have demonstrated substantial variability in end-of-life practices, with lower rates of treatment limitation observed in more religious societies and in regions with different cultural norms regarding autonomy and family involvement [2,11]. These differences suggest that religiosity does not operate in isolation but is embedded within broader systems of meaning, social expectations, and institutional practices. More recent European ICU data further indicate that temporal changes in end-of-life practice were not fully explained by patient or physician religious affiliation [10]. Over the same period, communication and family involvement in end-of-life decision-making also evolved, underscoring that changes in ICU practice occur within broader clinical and relational contexts.

A particularly noteworthy finding of this review is the association between clinician-provided spiritual care and end-of-life treatment patterns. In observational evidence, attention to patients’ spiritual needs was associated with less aggressive care and greater use of palliative approaches [27]. This is consistent with consensus recommendations emphasizing the importance of spiritual care within person-centered palliative care [4]. However, because the available evidence is observational, these findings should not be interpreted as demonstrating that spiritual care itself causes changes in treatment intensity or decision-making outcomes. From a relational perspective, spiritual care may provide a context in which patients and families can address existential concerns, although the effects of structured spiritual-care interventions require prospective evaluation.

The results of this review have important clinical implications. First, they underscore the need for clinicians to recognize and explore patients’ and families’ spiritual and religious beliefs as part of the decision-making process. Second, they suggest that training in spiritual care and communication skills may help clinicians navigate ethically complex situations and reduce variability in practice. Finally, they highlight the importance of culturally sensitive approaches that take into account the broader social context in which decisions are made. In this context, clinical competence may be understood not only in terms of technical expertise but also as the capacity to remain present in situations characterized by uncertainty, emotional intensity, and existential vulnerability.

Spirituality and religiosity do not simply influence decisions; they provide interpretive frameworks through which the meaning of life, suffering, and dying [28] is negotiated. In this sense, end-of-life decision-making in ICU settings may be understood not only as a clinical or ethical process but as a fundamentally existential process. *Clinical Implications for ICU Practice*

The findings of this review have important implications for clinical practice in intensive care units, where end-of-life decisions frequently occur under conditions of uncertainty, emotional distress, and limited time. Because spirituality and religiosity influence how patients, families, and healthcare professionals interpret illness, suffering, hope, and death, these dimensions should be regarded as integral components of person-centered critical care rather than peripheral or optional considerations. The principal practice implications arising from the synthesis are summarized in Table 4.

**Table 4:** Clinical implications for integrating spirituality and religiosity into ICU end-of-life care Abbreviation: ICU, intensive care unit Note: These clinical implications derive from the narrative synthesis of predominantly observational and qualitative evidence and should be interpreted as practice considerations rather than evidence of intervention effectiveness.

Clinical domain	Implication for ICU practice	Practical application
Spiritual assessment	Spiritual and religious beliefs should be explored as part of person-centered end-of-life decision-making rather than inferred from religious affiliation alone.	Use brief validated assessment approaches and open-ended questions to identify sources of meaning, beliefs, values, and existential concerns that may influence treatment preferences.
Family conferences	Structured family meetings can integrate medical information with the values, beliefs, and expectations shaping surrogate decision-making.	Create explicit space for discussion of spiritual or existential concerns during goals-of-care and end-of-life conversations.
ICU nursing	Nurses are well positioned to identify spiritual distress and tensions between clinical recommendations and family beliefs because of their continuous bedside presence.	Strengthen education in spiritual assessment and communication and incorporate nursing observations into multidisciplinary discussions.
Clinical psychology	Psychological support can help patients, families, and clinicians manage uncertainty, anticipatory grief, moral distress, and the emotional burden of end-of-life decisions.	Include clinical psychologists in complex cases involving decisional conflict, emotional distress, or difficulty tolerating uncertainty and loss.
Spiritual care providers	Chaplains and trained spiritual-care professionals can support meaning-making and existential coping beyond the provision of religious rituals.	Facilitate timely referral when spiritual distress, religious concerns, or existential questions become clinically relevant.
Advance care planning	Earlier discussion of values, treatment preferences, and spiritual beliefs may reduce uncertainty when patients later lose decision-making capacity.	Incorporate spiritual and religious values into advance care planning alongside preferences concerning life-sustaining treatment and goals of care.
Culturally responsive care	The meaning and influence of spirituality and religiosity vary across individuals, cultures, and healthcare systems.	Avoid assumptions based on religious affiliation or cultural background and use individualized, respectful dialogue to explore each patient’s perspective.

Taken together, these implications support a broader understanding of high-quality intensive care in which clinical expertise is complemented by attention to patients' spiritual, relational, and cultural dimensions. Integrating spiritual assessment, interdisciplinary collaboration, family-centered communication, advance care planning, and culturally competent practice may strengthen shared decision-making and contribute to end-of-life care that is both ethically sound and genuinely person-centered. Accordingly, consideration of spiritual needs may be incorporated into multidisciplinary ICU care when relevant to patients’ and families’ values, alongside psychological, social, and ethical dimensions of comprehensive critical care. *Limitations* Several limitations should be acknowledged. The included studies were heterogeneous in terms of design, populations, and measurement of spirituality and religiosity, which limited the possibility of quantitative synthesis. In addition, most studies relied on self-reported measures, which may be subject to bias. The restriction to English-language publications may also have excluded relevant studies from non-English-speaking regions. Furthermore, the relatively small number of included studies highlights the need for further research in this field, particularly using standardized measures and longitudinal designs. *Future Research Directions*

The evidence synthesized in this review points to a clinically important field that remains conceptually and methodologically underdeveloped. A central priority is greater precision in the definition and measurement of spirituality and religiosity. Across the included studies, these constructs were operationalized in markedly different ways, ranging from religious affiliation and ecological country-level indicators to measures of intrinsic religiosity, spiritual coping, and perceived spiritual support. These constructs are related but not interchangeable. Future research would benefit from distinguishing more clearly among religious identity, intensity of religious involvement, spirituality, spiritual coping, and sources of existential meaning, using validated multidimensional measures where appropriate. Greater conceptual and measurement consistency would improve comparability across studies and allow more precise interpretation of observed associations.

Methodological development is equally important. Much of the current evidence is cross-sectional or otherwise observational and therefore cannot establish temporal ordering or clarify whether spiritual and religious factors shape end-of-life preferences, change in response to critical illness, or both. Prospective and longitudinal designs are needed to examine how beliefs, spiritual needs, hope, acceptance, and treatment preferences evolve over the course of serious illness and ICU care. Particular attention should be given to the distinction between stated attitudes and actual clinical decisions, which may diverge under conditions of uncertainty, emotional distress, and time pressure. Where feasible, future studies should integrate patient-, family-, clinician-, organizational-, and healthcare-system-level variables within the same analytical framework.

The effects of spiritual care itself also require more rigorous evaluation. Existing findings suggest potentially meaningful associations with communication, decision-making, and the intensity of end-of-life care, but the evidence is predominantly observational and does not establish intervention effectiveness. Research should therefore move toward the evaluation of structured spiritual assessment, interdisciplinary models of spiritual care, and communication-based interventions through pragmatic trials, implementation studies, and mixed-methods designs. Relevant outcomes should extend beyond treatment limitation to include decisional conflict, concordance between expressed values and care received, quality of communication, moral distress among healthcare professionals, family experience, and bereavement-related outcomes.

Finally, future research should give greater attention to surrogate decision-making and to the cultural and relational contexts in which end-of-life decisions occur. In the ICU, patients are often unable to speak for themselves, placing families in situations where religious beliefs, uncertainty, grief, responsibility, and cultural expectations converge. Longitudinal qualitative and mixed-methods research may help clarify how these processes unfold over time and how they influence both decision-making and subsequent psychological adjustment. Multinational studies using harmonized measures would further help distinguish broadly shared patterns from those that are specific to particular religious traditions, healthcare systems, or cultural norms. Taken together, the field would benefit from moving beyond the treatment of spirituality and religiosity as static demographic characteristics and examining them instead as dynamic, relational, and context-dependent dimensions of how patients, families, and healthcare professionals confront serious illness, uncertainty, dying, and death.

## Conclusions

This systematic review indicates that spirituality and religiosity are important, context-dependent dimensions of end-of-life decision-making in ICU settings and are associated with differences in attitudes, ethical perceptions, treatment preferences, and reported clinical practices across patients, families, and healthcare professionals. Across several included studies, greater religious involvement or religiosity was associated with preferences for more aggressive, life-prolonging interventions and greater reluctance to withdraw life-sustaining treatment. At the same time, spirituality could also facilitate acceptance of death and support comfort-focused or palliative approaches, depending on the meaning attributed to individual beliefs and the context in which they were expressed.

These associations appeared to vary across cultural, social, relational, and healthcare-system contexts. Observational evidence further suggests that clinician-provided spiritual care and attention to spiritual concerns may be associated, in some settings, with less aggressive end-of-life care and greater concordance between care and patients’ expressed values. These findings, however, should not be interpreted as evidence that spiritual care itself causes improved clinical or decision-making outcomes. Prospective intervention and implementation studies are needed to establish whether structured approaches to spiritual care produce measurable benefits.

Clinicians should therefore remain attentive to spiritual and religious concerns when these are relevant to patients’ and families’ values, treatment preferences, and goals of care. Training in communication and spiritual assessment may support more sensitive and individualized end-of-life discussions, although the effectiveness of such approaches requires further evaluation. Future research should prioritize prospective, longitudinal, and intervention-based designs using conceptually precise and validated measures that distinguish religiosity, spirituality, religious affiliation, and spiritual coping.

Overall, recognizing spirituality and religiosity as potentially meaningful dimensions of critical illness may contribute to end-of-life decision-making that is more responsive to individual values, culturally informed, and person-centered.

## Appendices

Complete electronic search strategies

A comprehensive literature search was conducted in PubMed/MEDLINE, Scopus, and Web of Science. The final searches were completed on January 15, 2026. The database-specific strategies combined terms related to spirituality and religiosity, end-of-life decision-making, intensive and critical care, and adult populations, while excluding pediatric populations. No additional database filters or limits were applied beyond the terms incorporated into the search strategies.

PubMed/MEDLINE

Final search date: January 15, 2026 Records retrieved: 285 Additional database filters/limits: None

Search strategy: (spirituality OR religiosity OR religion OR religious OR "spiritual well-being" OR "Spirituality"[MeSH] OR "Religion"[MeSH] OR "Religion and Psychology"[MeSH]) AND ("end of life" OR "end-of-life" OR "EOL" OR "end of life care" OR "end of life decisions" OR "decision making" OR "treatment decisions" OR "End of Life Care"[MeSH] OR "Treatment Refusal"[MeSH] OR "Withholding Treatment"[MeSH] OR "Withdrawal of Treatment"[MeSH] OR "Do Not Resuscitate Orders"[MeSH]) AND (ICU OR "intensive care" OR "critical care" OR "critical illness" OR "Critical Illness"[MeSH] OR "Critical Care"[MeSH] OR "Intensive Care Units"[MeSH]) AND (adult OR "Adult"[MeSH]) NOT (infant OR newborn OR neonate OR neonatal OR pediatrics OR paediatrics OR child OR children OR adolescent OR "Infant"[MeSH] OR "Infant, Newborn"[MeSH] OR "Child"[MeSH] OR "Adolescent"[MeSH])

Scopus

Final search date: January 15, 2026 Records retrieved: 225 Additional database filters/limits: None

Search strategy: TITLE-ABS-KEY(spirituality OR religiosity OR religion OR religious OR "spiritual well-being" OR "Spirituality" OR "Religion" OR "Religion and Psychology") AND TITLE-ABS-KEY("end of life" OR "end-of-life" OR "EOL" OR "end of life care" OR "end of life decisions" OR "decision making" OR "treatment decisions" OR "End of Life Care" OR "Treatment Refusal" OR "Withholding Treatment" OR "Withdrawal of Treatment" OR "Do Not Resuscitate Orders") AND TITLE-ABS-KEY(ICU OR "intensive care" OR "critical care" OR "critical illness" OR "Critical Illness" OR "Critical Care" OR "Intensive Care Units") AND TITLE-ABS-KEY(adult OR "Adult") AND NOT TITLE-ABS-KEY(infant OR newborn OR neonate OR neonatal OR pediatrics OR paediatrics OR child OR children OR adolescent OR "Infant" OR "Infant, Newborn" OR "Child" OR "Adolescent")

Web of Science

Final search date: January 15, 2026 Records retrieved: 118 Additional database filters/limits: None

Search strategy: (spirituality OR religiosity OR religion OR religious OR "spiritual well-being" OR "Spirituality" OR "Religion" OR "Religion and Psychology") AND ("end of life" OR "end-of-life" OR "EOL" OR "end of life care" OR "end of life decisions" OR "decision making" OR "treatment decisions" OR "End of Life Care" OR "Treatment Refusal" OR "Withholding Treatment" OR "Withdrawal of Treatment" OR "Do Not Resuscitate Orders") AND (ICU OR "intensive care" OR "critical care" OR "critical illness" OR "Critical Illness" OR "Critical Care" OR "Intensive Care Units") AND (adult OR "Adult") NOT (infant OR newborn OR neonate OR neonatal OR pediatrics OR paediatrics OR child OR children OR adolescent OR "Infant" OR "Infant, Newborn" OR "Child" OR "Adolescent")

Search Yield

The three database searches identified 628 records in total: 285 from PubMed/MEDLINE, 225 from Scopus, and 118 from Web of Science. After removal of 108 duplicate records, 520 records remained for title and abstract screening.
